# Effectiveness of Personal Protective Equipment and Oseltamivir Prophylaxis during Avian Influenza A (H7N7) Epidemic, the Netherlands, 2003

**DOI:** 10.3201/eid1610.091412

**Published:** 2010-10

**Authors:** Dennis E. te Beest, Michiel van Boven, Marian E.H. Bos, Arjan Stegeman, Marion P.G. Koopmans

**Affiliations:** Author affiliations: Utrecht University, Utrecht, the Netherlands (D.E. te Beest, M.E.H. Bos, A. Stegeman);; National Institute of Public Health and the Environment, Bilthoven, the Netherlands (D.E. te Beest, M. van Boven, M.P.G. Koopmans)

**Keywords:** Avian influenza, zoonoses, poultry, human infection, personal protective measures, antiviral, viruses, the Netherlands, research

## Abstract

We analyzed the effectiveness of personal protective equipment and oseltamivir use during the 2003 avian influenza A (H7N7) epidemic in the Netherlands by linking databases containing information about farm visits, human infections, and use of oseltamivir and personal protective equipment. Using a stringent case definition, based on self-reported conjunctivitis combined with a positive hemagglutination-inhibition assay, we found that prophylactic treatment with oseltamivir significantly reduced the risk for infection per farm visit from 0.145 (95% confidence interval [CI] 0.078–0.233) to 0.031 (95% CI 0.008–0.073). The protective effect was ≈79% (95% CI 40%–97%). These results are comparable with the reported effect of prophylactic treatment with oseltamivir on human seasonal influenza. No significant protective effect was found for use of respirators or safety glasses, possibly because of limitations of the data.

## Abstract 2

TOC Summary: Only oseltamivir use significantly reduced the risk for human infection.

Avian influenza A viruses are considered a threat to public health because they may result in new human influenza A strains. Thus, knowledge about preventing human infections with avian influenza viruses is essential. In 2003, a devastating epidemic caused by an avian influenza virus of subtype H7N7 occurred among the poultry sector of the Netherlands (*1*). During this epidemic, an unexpectedly high number of persons reported illness that appeared to be associated with subtype H7N7 infection after they were exposed to infected poultry (*2*); 1 veterinarian died of acute respiratory distress syndrome (*3*). Previous reports have documented transmission between poultry, between humans, and from poultry to humans (*1**,**4**–**8*). In this report, we extend earlier work by analyzing the effect of personal protective measures on poultry-to-human transmission. Specifically, we investigated the effects of use of respirators and safety glasses and the prophylactic use of oseltamivir on the risk for infection during depopulation of infected farms. Our quantitative estimates of the effect of personal protective measures can guide efforts to prevent human infections with avian influenza.

## Materials and Methods

### Data

Immediately after the epidemic, the National Institute of Public Health and the Environment of the Netherlands sent a questionnaire to 1,747 persons, of whom 872 (49.9%) responded. Response was lowest among persons who actively handled the culling of poultry (*9*). Of the 872 persons who responded, 450 could be linked to a farm-visits database kept during the epidemic that contained information about who had visited which farm on which date for what reason. Of these 450 persons, 194 had been actively involved in hands-on culling during the depopulation; activities included catching live poultry and picking up dead poultry. Because this group had the highest exposure (*4*), it was used to analyze the effect of personal preventive measures.

The questionnaire asked for information about symptoms of infection of the eyes, from which a self-reported conjunctivitis result was derived as an outcome measure for subtype H7N7 infection. The presence of >2 of the following eye symptoms was classified as conjunctivitis: redness, tearing, itchiness, pain, burning, purulence, or sensitivity to light. Blood samples were collected from survey respondents 3 weeks after possible exposure and tested by a hemagglutination-inhibition (HI) assay for antibodies by using a modified cutoff based on validation studies of persons known to be infected and of nonexposed controls (*10*). The serologic result was available in addition to the self-reported conjunctivitis information.

We used a case definition that combined both outcomes, i.e., a person needed to self-report conjunctivitis and have a positive HI assay result. For sensitivity analysis, both the conjunctivitis and the serologic result were used in separate case definitions.

The survey also contained information about prophylaxis with a neuraminidase inhibitor (oseltamivir [Tamiflu; Roche, Basel, Switzerland]) and the use of personal protective equipment (PPE [safety glasses and respirator]). Beginning on March 14, prophylaxis with oseltamivir (75 mg daily) was prescribed to persons in contact with potentially infected poultry (2 weeks after the first infection was diagnosed and 10 days after culling began). Continuation of oseltamivir treatment was recommended until 2 days after possible exposure. The subtype H7N7 strain that circulated was susceptible to oseltamivir (*2*). To determine oseltamivir use, persons were asked the following questions: 1) Were you prescribed oseltamivir by a medical doctor? (yes/no). 2) If oseltamivir was prescribed, when was it prescribed and when did you stop using it? 3) How often did you fail to take a capsule? Persons who were not prescribed oseltamivir were classified as “did not use.” On the basis of the period of use stated in the survey, persons who were prescribed oseltamivir were classified per visit as “used” or “did not use.” Within the used category, persons who missed <3 capsules were classified as consistent users; if more capsules were missed, they were classified as inconsistent users.

**Figure 1:**
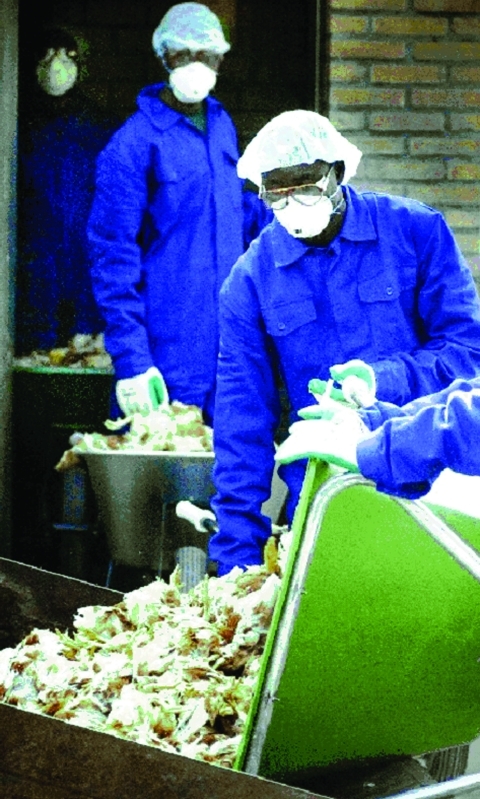
Poultry worker wearing respirator and safety glasses, the Netherlands, 2003.

PPE (respirator and safety glasses) were provided during the entire epidemic to persons involved in the depopulation. The Dutch Food and Consumer Product Safety Authority provided the PPE and supervised its use. For each visit, workers received new PPE. They were instructed how to use the PPE but received no extensive training. For both respirators and safety glasses, workers were asked the following questions: 1) Did you use respirators/safety glasses (yes/no)? 2) How often did you use respirators/safety glasses (always, almost always, sometimes, almost never, never)? 3) How often did you not use respirators/safety glasses? From responses to these questions, we classified persons into 3 categories: used, sometimes used, and did not use. Persons who had either always or almost always used respirators or safety glasses and who stated that they had not missed using them more than twice were placed in the “used” category. Persons who had not used them were classified as did not use, and all remaining persons with answers were classified as sometimes used. For safety glasses and respirators, information was available only about their use during the whole epidemic and not per visit. We therefore assumed that a person’s use of a respirator and safety glasses did not change during the epidemic. The respirators provided were type FFP2 (US equivalent N95, Figure 1), which protected both nose and mouth and were all the same size. Safety glasses covered only the front of the eyes and were open above, below, and on both sides of the eyes (Figure 1). They were effective against splashes but not against dust. No information was available about the overall health of the workers.

### Statistical Analysis

The probability of becoming infected during a visit was calculated from the number of visits each person had made and whether each person was infected (according to the case definitions). The analysis comprised only visits to farms with infected poultry. During each visit, a person could either escape infection or become infected. The probability of each sequence of events could be added, from which the probability of infection per visit could be estimated by maximum likelihood (Technical Appendix). The dependent variable in this analysis was the case definition, i.e., whether a person was infected. The probability of infection was calculated according to the categorical level of personal protection used, e.g., use of oseltamivir or not, as independent variables. We calculated confidence intervals (CIs) using profile likelihood methods (*11*). Statistical testing was performed by using likelihood ratio tests (*11*).

For oseltamivir, risk for infection was calculated per group and then aggregated over the groups of persons who had and had not used oseltamivir (Table 1, Table 2). Use of respirators, safety glasses, and oseltamivir were classified into the earlier described groups. We then calculated the risk for infection for each combination of groups, e.g., always used respirators/sometimes used safety glasses (Table 3, Table 4). Although onset date of infection was unknown, the likelihood function took into account that a person could become infected only once and that infection could occur at any farm visit.

**Table 1:** Number of infections and use of oseltamivir prophylaxis during avian influenza A (H7N7) epidemic, on the basis of combined conjunctivitis and serology-based case definition, the Netherlands, 2003*

Oseltamivir use	No. persons			No. visits	
	Infected	Total		By infected persons	Total†
Not prescribed	4	16		6	26
Prescribed but not used during visit	9	34		17	64
Prescribed and used consistently	4	41		6	85
Prescribed and used inconsistently	2	18		7	39
Prescribed and consistency of use unknown	2	7		2	18
Prescribed but period of use unknown	2	29		5	64

*Data resulted from a survey conducted among poultry workers after the epidemic. †Because visits were classified into groups, persons may be in 2 categories (prescribed/not used and prescribed/used).

**Table 2:** Risk for infection with avian influenza A (H7N7) with different levels of oseltamivir use, on the basis of combined conjunctivitis and serology-based case definition, the Netherlands, 2003*

Oseltamivir use	Risk† (95% CI)
Not used	0.145 (0.078–0.233)
Not prescribed	0.161 (0.052–0.336)
Prescribed but not used during visit	0.138 (0.061–0.246)
Used	0.031 (0.008–0.073)
Prescribed and used consistently	0.015 (0.0008–0.0630)
Prescribed and used inconsistently	0.049 (0.004–0.157)
Prescribed and consistency of use unknown	0.068 (0.004–0.258)
Unknown	0.019 (0.001–0.080)
Prescribed but period of use unknown	0.020 (0.001–0.080)

*CI, confidence interval. †Probability of infection per visit.

**Table 3:** Number of infections and use of respirator and safety glasses during avian influenza A (H7N7) epidemic, on the basis of combined conjunctivitis and serology-based case definition, the Netherlands, 2003*

Respirator used	Safety glasses used	No. persons			No. visits	
		Infected	Total		By infected persons	Total
Always	Always	1	19		3	44
	Sometimes	5	19		13	53
	Not used	4	27		8	45
Sometimes	Always	–	–		–	–
	Sometimes	3	17		11	58
	Not used	2	22		3	45
Not used	Not used	3	4		4	8
Unknown	Unknown	1	22		1	43

*Data resulted from a survey conducted among poultry workers after the epidemic. –, no data in this group.

**Table 4:** Risk for infection with avian influenza A (H7N7) with different levels of respirator and safety glasses use, on the basis of combined conjunctivitis and serology-based case definition, the Netherlands, 2003*

Respirator used	Safety glasses used	Risk† (95% CI)
Always	Always	0.023 (0.001–0.099)
	Sometimes	0.079 (0.018–0.188)
	Not used	0.093 (0.029–0.204)
Sometimes	Always	–
	Sometimes	0.056 (0.014–0.14)
	Not used	0.045 (0.007–0.133)
Not used	Not used	0.408 (0.119–0.755)
Unknown	Unknown	0.023 (0.001–0.099)

*CI, confidence interval; –, no data in this group. †Probability of infection per visit.

## Results

The 194 persons analyzed together had made 458 active culling visits. A conjunctivitis result was available for 193 persons (36 positive), a serologic result for 131 persons (81 positive), and a combined conjunctivitis/serology result for 130 persons (19 positive). Mean age of the study population was 40 years (range 18–64 years). Most (110) persons were veterinarians. Ninety percent of the study population was male. Persons made an average of 2.4 (range 1–9) active culling visits.

### Oseltamivir

Oseltamivir had been prescribed for 159 persons; 35 stated that oseltamivir had not been prescribed. Forty-five persons for whom oseltamivir had been prescribed did not use it during some of their visits. For the group of 130 (combined case definition), oseltamivir was prescribed for 114 and not prescribed for 16; for 34 persons, oseltamivir was prescribed but not used.

**Figure 2:**
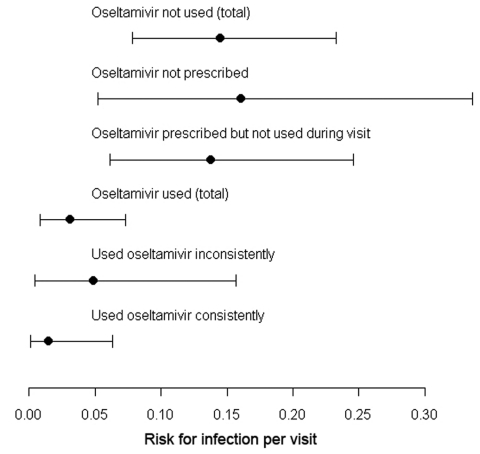
Point estimates and 95% confidence intervals of the risk for infection per visit in relation to oseltamivir use for the combined serology/conjunctivitis case definition, the Netherlands, 2003.

The estimated risk for infection per visit without use of oseltamivir was 0.145 (95% CI 0.078–0.233). This risk dropped significantly to 0.031 (95% CI 0.008–0.073; p = 0.005) per visit when oseltamivir was used (Table 2; Figure 2). When calculated over the infection probabilities, oseltamivir use had a protective effect of 79% (95% CI 40%–97%; relative risk [RR] 0.21, 95% CI 0.03–0.60). The risk for infection seemed to increase when oseltamivir was used inconsistently, but this risk did not reach significance.

Results with case definitions that used either the serologic results or self-reported conjunctivitis differed slightly. With the serologic result, the estimated risk for infection without oseltamivir use was 0.513 (95% CI 0.370–0.656). This risk dropped to 0.274 (95% CI 0.157–0.418; p = 0.01) with oseltamivir use (Table 5, Table 6), which resulted in a protective effect of 46% (95% CI 19%–68%; RR 0.54, 95% CI 0.32–0.81). With self-reported conjunctivitis used as case definition, the estimated risk for infection without oseltamivir use was 0.115 (95% CI 0.064–0.180). This risk dropped to 0.073 (95% CI 0.032–0.135; p = 0.08) with oseltamivir use (Table 5, Table 7), which resulted in a protective effect of 53% (95% CI 2%–82%; RR 0.47, 95% CI 0.18–0.98).

**Table 5:** Comparison of conjunctivitis and serologic result case definitions for number of infections and prophylactic oseltamivir use during avian influenza A (H7N7) epidemic, the Netherlands, 2003*

Oseltamivir use	Conjunctivitis			Serologic result	
	Persons†	Visits‡		Persons	Visits
Not prescribed	6/35	9/63		12/16	22/26
Prescribed but not used during visit	12/45	25/80		26/34	48/64
Prescribed and used consistently	7/54	9/113		24/41	54/85
Prescribed and used inconsistently	5/26	13/65		11/18	24/39
Prescribed and consistency of use unknown	4/9	8/23		4/7	11/18
Prescribed but period of use unknown	9/47	15/109		17/30	40/68

*Data resulted from a survey conducted among poultry workers after the epidemic. †No. persons infected/total no. persons. ‡No. visits by infected persons/total no. visits. Because visits were classified into groups, persons may be in 2 categories (prescribed not used and prescribed used).

**Table 6:** Risk for infection with avian influenza A (H7N7) with different levels of oseltamivir use, on the basis of serologic result as case definition, the Netherlands, 2003*

Oseltamivir use	Risk† (95% CI)
Not used	0.513 (0.370–0.656)
Not prescribed	0.653 (0.398–0.870)
Prescribed but not used during visit	0.446 (0.277–0.623)
Used	0.275 (0.182–0.381)
Prescribed and used consistently	0.294 (0.178–0.432)
Prescribed and used inconsistently	0.281 (0.117–0.493)
Prescribed and consistency of use unknown	0.181 (0.016–0.502)
Unknown	0.274 (0.157–0.418)
Prescribed but period of use unknown	0.274 (0.157–0.419)

*CI, confidence interval. †Probability of infection per visit.

**Table 7:** Risk for infection with avian influenza A (H7N7) with different levels of oseltamivir use, on the basis of conjunctivitis as case definition, the Netherlands, 2003*

Oseltamivir use	Risk† (95% CI)
Not used	0.115 (0.064–0.180)
Not prescribed	0.098 (0.040–0.189)
Prescribed but not used during visit	0.127 (0.055–0.227)
Used	0.054 (0.026–0.097)
Prescribed and used consistently	0.041 (0.012–0.091)
Prescribed and used inconsistently	0.052 (0.010–0.135)
Prescribed and consistency of use unknown	0.149 (0.029–0.360)
Unknown	0.073 (0.032–0.135)
Prescribed but period of use unknown	0.073 (0.032–0.135)

*CI, confidence interval. †Probability of infection per visit.

We could not analyze interactions between use of oseltamivir and PPE because the resulting groups would have had too few visits. In the group that always used respirators and protective glasses and the group that sometimes used respirators and protective glasses, oseltamivir use was relatively equal (63%–74%). In the group that sometimes used respirators and did not use safety glasses, oseltamivir was used in 38% of the visits. In the group that did not use safety glasses or respirators, oseltamivir was used in 25% of the visits. These findings indicate no strong correlation.

### Respirators and Safety Glasses

Persons generally were more inclined to use respirators than safety glasses (Table 3, Table 8). Persons who always used safety glasses also used a respirator. Only a small number of persons stated they had used no respirator and no safety glasses.

**Table 8:** Comparison of conjunctivitis and serologic result case definitions for number of infections and use of respirators and safety glasses during avian influenza A (H7N7) epidemic, the Netherlands, 2003*

Use of respirator	Use of safety glasses	Conjunctivitis			Serology	
		Persons†	Visits‡		Persons	Visits
Always	Always	3/28	5/60		11/19	30/44
	Sometimes	7/25	16/76		12/19	38/53
	Not used	7/38	14/65		19/27	32/45
Sometimes	Always	–	–		–	–
	Sometimes	4/25	13/84		10/18	40/62
	Not used	5/28	12/62		10/22	19/45
Not used	Not used	4/14	6/34		4/4	8/8
Unknown	Unknown	6/35	13/72		15/22	32/43

*Data resulted from a survey conducted among poultry workers after the epidemic. Totals of respirator and safety glasses use can be derived by adding up the separate groups. –, no data in this group. †No. infected persons/total no. persons. ‡No. visits by infected persons/total no. visits.

We gauged the effect of using safety glasses by comparing the risk for infection in persons with different safety glasses use within the group who always used respirators (Table 4). Within this group, risk for infection decreased with use of safety glasses; however, this trend was not significant. We gauged the effect of respirator use by comparing the risk for infection in persons with equal levels of safety glasses use but different respirator use (Table 4). Risk for infection was lower in persons who sometimes used a respirator than in persons who always used a respirator (within the groups that sometimes used or did not use safety glasses). Risk was higher for persons who had not used respirators than for those who had sometimes or always used them (within the group that had not used safety glasses). However, the group that had used neither respirators nor safety glasses was small. In both comparisons, the protective effect of respirators was not statistically significant. In the sensitivity analysis, results for which conjunctivitis and the serologic result were used as case definitions were similar to results with the combined case definition (Table 9).

**Table 9:** Comparison of conjunctivitis and serologic result case definitions for risk for infection with avian influenza A (H7N7) with different level of respirator and safety glass use, the Netherlands, 2003*

Use of respirator	Use of safety glasses	Risk† with conjunctivitis used as case definition (95% CI)	Risk† with serology used as case definition (95% CI)
Always	Always	0.051 (0.012–0.127)	0.338 (0.181–0.534)
	Sometimes	0.063 (0.017–0.144)	0.274 (0.125–0.471)
	Not used	0.115 (0.050–0.210)	0.494 (0.320–0.672)
Sometimes	Always	–	–
	Sometimes	0.050 (0.015–0.114)	0.222 (0.108–0.380)
	Not used	0.082 (0.027–0.173)	0.233 (0.118–0.383)
Not used	Not used	0.121 (0.039–0.261)	1 (0.463–1)
Unknown	Unknown	0.088 (0.035–0.171)	0.48 (0.296–0.676)

*CI, confidence interval; –, no data because no persons in this group sometimes used a respirator and always used safety glasses. †Probability of infection per visit.

## Discussion

Quantifications of the effect of prophylactic use of oseltamivir on the risk for infection with avian influenza A (H7N7) virus can be used to guide efforts to reduce human infections with avian influenza. The risk for infection per visit was remarkably high among persons who did not use prophylactic oseltamivir (0.145, 95% CI 0.078–0.233). Although significantly lower, the risk for infection per visit for persons who did use oseltamivir prophylactically was still considerable (0.031, 95% CI 0.008–0.073).

Given the lack of research on the prophylactic effect of oseltamivir use on avian influenza in humans, we compared our result with studies on human seasonal influenza. Our estimated protective effect of oseltamivir use (79%) compares with that reported for laboratory-confirmed symptomatic human seasonal influenza, which ranges from 68% to 90% (*12**–**17*). The protective effect estimated with the case definition based on the serologic result only (46%) is close to the range of 49%–68% found with laboratory-confirmed human influenza (*12**–**14*). This finding suggests that the specificity of the serologic outcome measure is high, even though in our study it has a relatively low cutoff (*10*). The standard for use of HI assays in serologic studies is a cutoff of 40. This cutoff was lowered on the basis of evidence from an epidemiologic study in which no serologic responses were found in any of the 89 known infected persons by using the standard criteria, but a high proportion had low-level antibody reactivity with high specificity, which has triggered some debate about the validity of this serologic approach. However, finding a significantly lower risk for persons that used oseltamivir and had antibody reactivity cannot be explained by nonspecific reactivity. Therefore, we conclude that, for some reason, subtype H7N7 infections do not provoke a strong immune response, possibly related to the ocular tropism. Since then, similar findings have been reported (*18**,**19*). Finally, the protective effect when conjunctivitis is used as case definition was 53% in our study, compared with 29% for human influenza based on influenza-like illness (*14*), which suggests a reasonable specificity of conjunctivitis as indicator for subtype H7N7 infection.

Considering the effect of oseltamivir in reducing the risk for infection, prophylactic treatment of all persons involved in depopulating farms may seem wise. However, oseltamivir is also the fallback drug used for treating patients with severe influenza, and resistance against oseltamivir has increased in seasonal influenza viruses (*20*). A decision regarding prophylactic use of oseltamivir in a future epidemic would need to account for the risk for infection, risk for resistance, and severity of the infection.

Although our results provided clear evidence that prophylactic use of oseltamivir is effective in reducing risk for infection, the results were less clear with regard to PPE. In fact, although use of safety glasses appeared to reduce the risk for subtype H7N7 infection, this effect was not significant. Considering that the main symptom of subtype H7N7 is conjunctivitis, safety glasses may protect the eyes to some extent against influenza infection. For respirators, we also did not find a clear protective effect. Persons who always wore respirators may have done work that exposed them more. For both respirators and safety glasses, people received a limited amount of training that perhaps led to ineffective use of PPE and unsafe removal of contaminated PPE. Respirators were available in only 1 size and thus may not have fit well. The safety glasses were effective against splashes but were open on all sides and were not effective against dust. Possibly the PPE used were not appropriate for the high-exposure work. The number of available visits per group was low, data were also of limited temporal resolution (because PPE use per visit was not available), and potential for a recall bias also existed. Because of these limitations, we cannot conclusively determine from this study that respirators and safety glasses do not provide a protective effect. Findings of an experimental situation (*21**,**22*) indicated that respirators are likely to modify exposure and thus risk for infection. In Norfolk, United Kingdom, in 2006, incomplete use of PPE (safety glasses and respirator) was associated with conjunctivitis and influenza-like illness in an outbreak of avian influenza A (H7N3) (*23*).

In our study, prophylactic use of oseltamivir greatly reduced risk for infection with avian influenza A (H7N7). However, even with oseltamivir use, risk for infection remains considerable. Oseltamivir use should be part of an integrated approach to reduce human exposure, together with the use and appropriate training of PPE.

## Supplementary Material

Technical Appendix Maximum Likelihood Method.
