# Supplementary material for: Effectiveness of Personal Protective Equipment and Oseltamivir Prophylaxis during Avian Influenza A (H7N7) Epidemic, the Netherlands, 2003
Source: Emerg Infect Dis. 2010 Oct;16(10):1562–8. doi: 10.3201/eid1610.091412 (PMC3294382; doi:10.3201/eid1610.091412)
Supplement: Technical Appendix — Maximum Likelihood Method. [file 09-1412-Techapp_2p.pdf]

Article DOI: 10.3201/eid1610.091412

Effectiveness of Personal Protective Equipment and Oseltamivir Prophylaxis during Avian Influenza A (H7N7) Epidemic, the Netherlands, 2003

Dennis E. te Beest, Michiel van Boven, Marian E.H. Bos, Arjan Stegeman,
and Marion P.G. Koopmans

Technical Appendix

Maximum Likelihood Method

Each visit is assigned a probability p_{ij}, which is the risk that person *i* becomes infected during visit *j*. Each person can of course become infected during any visit or escape infection. The entire sequence of possible events (e.g., person *i* infected during the first visit or person *i* infected during second visit) is summed over all persons and all visits according to equation 1. The risk for infection (p_{ij}) is then estimated by maximizing this equation:

$$L = \prod_{i \in P \setminus I} \prod_{j=1}^{n_i} (1 - p_{ij}) \cdot \prod_{i \in I} \sum_{j=1}^{n_i} p_{ij} \prod_{k=1}^{j-1} (1 - p_{ik})$$

where P is the set of all persons in the data, I is the subset of infected persons, and n_i is the number of visits made by person *i*. All p_{ij}s are classified into groups according to the levels of personal protection used. The levels of protection (i.e., oseltamivir, safety glasses, and respirators) are the independent variables in this analysis. The dependent variable is the case definition, i.e., regardless of whether someone is infected.

In a previous article, we classified persons with respect to their activities in the whole process leading to depopulation (*1*). Here we focus on the subset of persons who were active during depopulation. The estimated probabilities in the depopulation subset can be influenced by other visits. Someone involved in depopulation may also have made tracing visits. For this

reason (for a correct estimation) the other types of visits made (screening, indexing, tracing, cleaning assistance culling, biosecurity culling) also were included in the model. Risks for infection at these visits remained constant throughout the analysis. Results for these quantifications have been discussed in (*1*).

Reference

1. Bos MEH, te Beest DE, van Boven M, Robert M, Meijer A, Bosman A, et al. High probability of avian influenza virus (H7N7) transmission from poultry to humans active in disease control on infected farms. J Infect Dis. 2010;201:1390–6. PubMed
